# Editorial: Old drugs: confronting recent advancements and challenges

**DOI:** 10.3389/fphar.2025.1565890

**Published:** 2025-03-19

**Authors:** Anna Wiktorowska-Owczarek, Diego Iacono, Magdalena Jasińska-Stroschein

**Affiliations:** ^1^ Department of Pharmacology and Toxicology, Medical University of Lodz, Lodz, Poland; ^2^ Neuropathology Research, MidAtlantic Neonatology Associates (MANA)/Biomedical Research Institute of New Jersey (BRInj), Cedar Knolls, NJ, United States; ^3^ Neuroscience Research MANA, Department of Pediatrics, Atlantic Health System (AHS), Morristown, NJ, United States; ^4^ Neurodevelopmental Research Lab, Biomedical Research Institute of New Jersey (BRInj), Cedar Knolls, NJ, United States; ^5^ Department of Neurology, Thomas Jefferson University, Philadelphia, PA, United States; ^6^ Department of Biopharmacy, Medical University of Lodz, Lodz, Poland

**Keywords:** drug repositioning, old drugs-new uses, overlooked mechanisms, new therapeutic perspectives, safety aspects

## Introduction

Drug repurposing involves leveraging existing drugs or drug candidates, originally developed for other conditions, to address new therapeutic purposes or medical conditions. This strategy often draws from observations of unintended or off-target effects, including those initially seen as adverse or age-specific, which may reveal their potential to alleviate chronic, degenerative, or age-related conditions. The core characteristics of drug repurposing may be summarized as follows:

1. *Well-Known Safety*: Repurposed drugs are typically already tested for safety in humans, significantly reducing early-stage development risks.
2. *Streamlined Development*: These drugs can bypass initial drug discovery phases, proceeding directly to preclinical or clinical trials.
3. *Cost and Risk Red*uction: By skipping early development stages, repurposing lowers both financial costs and associated risks.
4. *Time Efficiency*: The process offers a quicker path to market, addressing unmet medical needs efficiently.

More recently, the search for repurposing candidates combines traditional and modern tools, including but not limited to computational analysis and database searches (i.e., utilizing public databases such as medical journals, regulatory agency archives, and patient advocacy group reports); newer experimental approaches (i.e., testing drug interactions in novel contexts); artificial intelligence and bioinformatics tools (i.e., advanced algorithms explore potential drug-protein interactions, integrating pharmacological data with genetics, pharmacogenomics, biological pathways, and diagnostic/prognostic insights).

For the above reasons, over the years, there has been an increasing demand for a more effective and cost-efficient drug discovery process. To do so and in addition to searching for. In review entirely new molecules to be included in therapies, drug repositioning is a dynamically developing area in the search for effective therapy for many diseases. This approach is actually particularly appealing since it saves time and resources and reduces the risk of failure or unexpected safety issues.

The aim of the Research Topic was to inspire you to present your research, but also to encourage the interested readers to review drugs whose scope of action has expanded over the years and, additionally, open up new points of entry in the treatment of various diseases. The Research Topic features six review articles, including one systemic review. Additionally, the Research Topic includes four original research papers.

In the original papers, commonly known drugs were analyzed, but contextualized in new applications. For example, Chen et al. searched for antiviral treatment for parainfluenza 3 (PIV3) illness. The authors explored an integrated drug repurposing method, including disease similarity and chemical similarity as multi-similarity analysis approaches, molecular docking and molecular dynamic simulation methods as structure-based screening approaches, and network proximity analysis, in which they quantified the network distance between the disease module of PIV3 and the drug targets to probe the potential anti-PIV3 drugs. Using the aforementioned method, they confirmed that oseltamivir is the best potential drug against PIV3. Therefore, they recommend considering oseltamivir for clinical application in children.

The next compound analyzed for a potential new indication was rosiglitazone, which is a thiazolidinedione used in the treatment of diabetes (Lehmann et al., 1995). Rosiglitazone has been reported to ameliorate high-fat diet-induced obesity in mice (Petrovic et al., 2010). As Li et al. showed, this antidiabetic drug promotes adipocyte browning by inhibiting autophagy.

More specifically, inhibition of autophagy by rosiglitazone increased p62 nuclear translocation and stabilized the PPARγ-RXRα heterodimer for the transcription of browning genes. These studies underlined the promising role of rosiglitazone in the treatment of obesity. Interestingly, a new challenge has been set for dexmedetomidine (DEX), namely, its participation in the protection against many degenerative conditions, including neurodegenerative diseases, by reducing oxidative stress. Indeed, DEX is a highly selective α2-adrenoceptors agonist, has a sedative, analgesic effect and anti-sympathetic properties (Keating, 2015). Lou et al. research has shown that DEX effectively reduces oxidative stress, avoids apoptosis and protects osteoblasts. The authors propose it for the prevention of bone defects, which could also be utilized though for other non-bone defects.

The last of the original research tested in the diabetic retinopathy (DR) the purine analogue 6-thioguanine (6TG), an old drug approved in the 60s to treat acute myeloid leukemia (AML) – Trotta et al. The authors concluded that 6TG revealed a marked anti-angiogenic activity in HUVECs exposed to high glucose and in mice with DR, and this activity was mediated by MC1R and MC5R retinal receptors.

In turn, review papers considered the possibility of repurposing effect of cardiovascular-metabolic drugs to prolong life (Barinda et al.). This systematic review mainly addressed to animal models (short-lived, obese or diabetic mice), where numerous cardiovascular, antidiabetic and lipid-lowering medications proved to successfully extend the lifespan. The authors also provide summary of ongoing clinical trial in repurposing cardiometabolic drug (metformin, omega-3 fatty acid, acarbose, and atorvastatin) for aging, expressing an expectation that the obtained findings will give the support for the utility of these medications in healthy subjects’ cardiovascular or neurological aging. The pleiotropic effects exerted by statins and beta-blockers gave the rationale to evaluate their possible complementary use in solid tumours, including breast, colorectal, lung, and prostate cancers (Braga et al.). The authors provided an update of the impact of these therapies on cancer treatment and surveillance, discussing the underlying mechanisms, and exploring their effects on the heart, contributing to the growing field of cardiooncology. The review of findings from retrospective analyses, and randomized clinical trials within the cancer continuum allowed the authors to conclude that β-blockers seemed to lead to better clinical outcomes for breast cancer, whereas statins were positively associated with greater outcomes in breast, colorectal and prostate treatment. Further evaluations of drug dosage, time of treatment and benefits of different classes of β-blockers and statins are needed, however.

Fatemi et al. provided a comprehensive review of research efforts, and examples of drugs repurposing in different types of gastrointestinal cancers, such as colorectal, pancreatic, and liver cancer. Moreover, the authors have addressed several barriers for drug repurposing for cancer therapy, including the legal, regulatory, patent issues and financial factors. In the work by Glajzner et al., the authors presented current data on the potential of drug repositioning across various therapeutic classes for the treatment of infectious diseases caused by multidrug-resistant bacteria. This review also explored the therapeutic possibilities that arise when these drugs are combined with antibiotics. Preclinical studies demonstrated considerable promises for drugs from a range of therapeutic areas, including oncology, psychiatry, and pain management. The review has shown that drug repositioning candidates for infectious diseases can impact bacterial metabolism and cellular structure, as well as damage their genetic material. However, authors emphasized the need for further research; this should exclude the risk that potential antibacterial candidates would induce resistance -either to the drug itself or to the antibiotics used in conjunction. In a mini-review by Huang et al., the protective effects of different anaesthetic agents in intestinal ischemia-reperfusion injury were presented.

The reviewed strategies of drug repurposing involved also exploring new therapeutic applications for anti-cancer drugs. Choi et al. suggested that they can be regarded as candidates for repurposing of Alzheimer’s disease (AD) treatments. In particular, the authors emphasized the role of epidermal growth factor receptor (EGFR) as an important molecular target for both cancer and AD. The EGFR pathway is a widely recognized oncogenic pathway for non-small cell lung cancer. In the current review the authors mainly focused on the possible linkage between EGFR upregulation and inhibition of cancer cell migration or promotion of AD pathology (e.g., production and deposition of the β-amyloid peptide, neuroinflammation, or cognitive function).

We are convinced that this Research Topic may offer a Research Topic of original and review manuscripts aiming to identify several selected drug candidates for specific repositioning treatments. Repurposing existing drugs with a well-known pharmacological and toxicological profile is an attractive method for quickly discovering new therapeutic indications. The off-label use of drugs for different diseases requires much less capital and time, and can hasten progress in the development of new drugs. Conversely, pharmaceutical industry seems to be, historically less interested in investing in further research with already approved or marketed drugs, and this may be direct consequence of the lack of economic incentives, regulatory protection or decreased chance for a new patent. Then, the potential novel indications, posologies or formulations for old medications are mainly studied in the independent clinical trials being conducted by research institutes, academia or collaborative groups. It is then clear that additional financial support is needed to confirm the possible results in terms of efficacy and safety of various but small I and II phase clinical studies, in large but still too expensive, time-consuming, and labor-intensive randomized controlled trials. Therefore some authors advocate for additional public funding as well as increased harmonization and centralization of such clinical research activities (Verbaanderd et al., 2021). This latter aspect becomes especially important in the light of the fact that in many circumstances the promises for repositioning old drugs from a wide range of therapeutic areas (even outside the original therapeutic area for which specific compounds were approved for) have been demonstrated in preclinical studies only, and further clinical trials are required to confirm their potential for such a clinical adoption, which in certain cases, could even provide higher efficacy, safety and increased wellbeing that initially thought (Aronson, 2007; Rechberger et al., 2025).
